# Education in Army Hospitals

**Published:** 1921-01-15

**Authors:** 


### EDUCATION IN ARMY HOSPITALS.

### By a Hospitals' Area Education Officer.

The wounded soldier in hospitals at home during the war was overwhelmed with every conceivable diversion—mental and physical. Everything was done to wean his mind from past disasters and to coax his body back to its normal functionings. Education at that time went steadily forward with convalescence. Soldiers with shattered brains and paralysed right arms had to be taught from the beginning again. One such man started by grunting when asked a question or shown his tunic or cap—quite incapable of speech. He left hospital just able to write his name in capitals and to read from an easy story-book. Other men were insatiable in their desire for refresher courses, and the balcony of one hospital found Russian, shorthand, and the micrometer gauge in adjacent beds.

### Injudicious Work.

In some cases, however, it was during this time that so much injudicious work was done, and many now realise that, although as a pastime some of it was of positive value, inasmuch as convalescence is always assisted by occupational work, the mischief was only apparent when the disabled ex-soldier tried to eke out his pension with a little home work. He at once found that the cost of materials and the lack of a favourable market, together with the rapidly evaporating regard for his services rendered, left the work almost always on his hands, or alternatively cut his profits to a minimum.

Where definite training schemes for a long period were taken up by (then) the Ministry of Pensions, the results, of course, were not the same—but the subject of this article is the much narrower one of the ordinary soldier in the ordinary hospital.

At that time every type of craftsmanship came into hospital, both intellectual and manual. Classes were held in subjects ranging from Hindustani to millinery and from higher mathematics to making soap-boxes, but now the post-war army has found a level, so that education on the one side becomes a matter of Army education certificates, and on the other a very little hand and eye training. An after-war lassitude seems to overhang the world's production, and nowhere is this more felt than with the soldier in hospital. He is mentally and physically war weary, and controlled ambition seems a very undesirable expenditure of energy.

### Must Training be Voluntary?

Army education higher authorities have firmly adhered to the principle that to a convalescent man all educational training must be voluntary, for unwelcome activity both intellectual and manual only dissatisfies the patient and retards convalescence. There were some popular classes, and curiously enough we found that glee-singing by choirs of patients worked wonders. Apart from any psychotherapeutical value the singing had, it was immensely enjoyed, but after all its educational utility was small, and a hard-hearted Treasury insisted upon cold facts and returns. This was our greatest trial.

The third, second, first, and special Army education certificates were obtained in a few cases, and a very great many examining bodies of Universities and trade institutions accepted the "special" as equivalent to their own matriculation and preliminary qualifying examinations respectively, but even then the ding-dong classes were very small.

We tackled the men over their dinners, we spoke to them in the wards before they were visited in the morning. We ran cinemas and talked shop in the intervals, and still the ding-dong work lagged, and the manual work seemed to have no appeal. Photographical excursions—visits to places of historical interest—a very courteous superintendent of the Naval Dockyard at Portsmouth, all broadened the men's outlook, but here again the money was necessarily the problem; and the main issue, the progress reports.

### Training Must Have Market Value.

In this particular hospital I have striven hard to provide for the men some experience which will be a definite marketable asset whether leaving or continuing in the service. Accordingly we have steadfastly set ourselves to teach the man how to eke out his pension or his civil employment. Eight fowls—second strain White Wyandottes and Black Leghorns—were given to me by some ladies interested, and a poultry house and run built in the carpenter's shop by the men themselves on a most up-to-date design provided by a well-known poultry expert. With two patients at a time taking on the feeding and cleaning-up for a fortnight, with demonstrations to the rest of the poultry class three times a week, we made the hens pay handsomely. Our average has been six eggs a day until moulting-time, and these eggs were sold to the officers' mess and the like, at market prices. Every man who has been through that class knows how to feed and keep eight hens, and how to make them pay. That is my idea of training in hospital.

The futility must be apparent of instructing a soldier how to run a large poultry farm intensively on modern lines and then withholding from him the necessary capital. Poultry farming on a large scale requires large capital and thoroughly expert knowledge to make it pay.

Similarly with our basket-making and our carpentry. It has never been desired to show the soldier how to become a fully qualified carpenter, or to show him how to make a living by making baskets. Teach the patient how to make his pipe-rack or his shelves for the kitchen, or how to unhinge a door that jams and plane down the offending wood and reswing the door to make it a "good job," and our work is done. Teach his next-door neighbour to make a cane napkin ring, or a cane flower-pot stand, or a shopping basket, and we have put some money in his pocket.

We have, therefore, tried to steer clear of controversial matters relating to trades unions and apprenticeships, and the manual training in the area under my own supervision is modestly utilitarian only. Small in compass the training certainly is, but all knowledge, combined with experience, should be productive, and if in these days of industrial non-productiveness the small seed can be sown of a progressive self-reliance, is it unreasonable to hope that in its season the full fruition of a sane commonwealth will bring back this country to a desperately needed renaissance of productive activity?

